# In Vivo Evaluation of Miconazole-Nitrate-Loaded Transethosomal Gel Using a Rat Model Infected with *Candida albicans*

**DOI:** 10.3390/ph17050546

**Published:** 2024-04-24

**Authors:** Zara Asghar, Talha Jamshaid, Usama Jamshaid, Asadullah Madni, Naheed Akhtar, Manar O. Lashkar, Heba A. Gad

**Affiliations:** 1 Department of Pharmaceutics, Faculty of Pharmacy, The Islamia University of Bahawalpur, Bahawalpur 63100, Pakistan; zaraasghar21m@gmail.com (Z.A.); asadpharmacist@hotmail.com (A.M.); naheedakhtar96@yahoo.com (N.A.); 2 Department of Pharmaceutics, Faculty of Pharmacy, Strasbourg University, 67084 Strasbourg, France; usama.jamshaid042@gmail.com; 3 Department of Pharmacy Practice, Faculty of Pharmacy, King Abdulaziz University, Jeddah 21589, Saudi Arabia; mlashkar@kau.edu.sa; 4 Department of Pharmaceutics and Industrial Pharmacy, Faculty of Pharmacy, Ain Shams University, Cairo 11566, Egypt; 5 Department of Pharmaceutical Sciences, Pharmacy Program, Batterjee Medical College, Jeddah 21442, Saudi Arabia

**Keywords:** miconazole nitrate, antifungal efficacy, transethosomal gel, in vivo evaluation, histopathology, hematology, public health

## Abstract

Miconazole nitrate (MCNR), an antifungal drug, is used to treat superficial infections. The objective of the current study was to assess the antifungal effectiveness of MCNR-loaded transethosomal gel (MNTG) against *Candida albicans* in an in vivo rat model. The outcomes were compared with those of the miconazole nitrate gel (MNG) and marketed Daktarin^®^ cream (2%) based on histopathological and hematological studies. The results of the skin irritation test revealed the safety profile of the MNTG. The MNTG demonstrated the greatest antifungal activity in the histological analysis and the visible restoration of the skin, and the rats revealed an apparent evidence of recovery. Compared to the untreated group, the treated group’s lymphocyte and white blood cells counts increased, but their eosinophil counts decreased. In conclusion, MNTG exhibited the greatest antifungal activity, which might be connected to the improved skin permeability of the transethosome’s nanosized vesicles. Therefore, it could be considered a promising carrier for topical usage and the treatment of cutaneous candidiasis. More clinical research needs to be performed in order to demonstrate its effectiveness and safe usage in humans.

## 1. Introduction

Fungal infections are one of the most serious dermatological disorders carried by different types of pathogenic fungi. Over 25% of the world’s population suffers from fungal infections [1]. The root cause of the fungal infection identified as candidiasis is a fungus that looks like yeast called *Candida*. Some kinds of *Candida* can infect people; *Candida albicans* is the most widespread. Without causing any distress, *Candida* usually resides on the skin as well as in bodily parts like the throat, mouth, vagina, and gut. When *Candida* overgrows or invades internal organs like the kidney, heart, or brain, for instance, or the circulation, it may result in infections [2]. *Candida* infection is referred to as candidiasis or candidosis [3]. Currently, one of the most challenging diseases to treat in humans is fungal infection. Patients with severe underlying conditions (e.g., cancer) or immunocompromised patients are at a greater risk of developing invasive and fatal fungal infections [4]. More than 300 million individuals worldwide are believed to have a deadly fungal infection that has killed 1.4 million people a year, with *Aspergillus* and *Candida* species accounting for the majority of infections [5]. Although superficial mycosis is the most widespread fungal infection [6].

Antifungal medications of many types are offered to treat invasive cutaneous as well as fungal infections on the skin. Only a few types of antifungal medications are available despite an increase in the occurrence of fungal infections, predominantly in immunocompromised individuals, and they show only little success in the management of infections that pose a serious threat to life. Azoles are some of these medications [7].

Treatment for candidiasis is provided by the broad-spectrum antifungal drug miconazole nitrate (MCNR), which belongs to the imidazole group. MCNR has minimal systemic effectiveness because of its inadequate water solubility (less than 1 µg/mL) and extensive hepatic transformation [8]. The drug’s systemic delivery can also cause patients to experience some of the renowned adverse pharmacological reactions, such as nausea, abdominal pain, bloating, and vomiting. The mechanism of action of MCNR depends on the suppression of ergosterol production, which leads to the lysis of fungal cell membranes and the suppression of peroxidase, which causes a buildup of peroxide inside the cell and cell death [9]. Due to inadequate skin penetration, the topical administration of MCNR is troublesome in the treatment of cutaneous disorders. To evade this problem and formation for inadequate permeability, conventional formulations have to be taken at higher doses.

Lipid vesicles have recently received a lot of focus as carriers for topical medications because they are able to circumvent the skin’s innate barrier functions [10]. The incredibly flexible vesicles known as transethosomes have a bilayer structure. They can readily squeak across the intracellular lipid of the stratum corneum to infiltrate the skin and defeat the barrier function [11]. Transethosomes are an innovative method of drug administration that is frequently used for topical treatments. Transethosomes are vesicular carriers that are flexible and soft. Transethosomes are made up of ethanol, edge activators, and phospholipids. Transethosomes are spherical and uneven, and have a higher degree of elasticity and skin permeation/penetration. This is attributed to the lipid bilayer of these vesicle changes as a result of the interaction between the ethanolic effect and an edge activator or surfactant [12]. Different permeability enhancers and edge activators are employed to generate transethosomal systems with improved characteristics [13].

MCNR is suggested to be formulated in a topical dosage form in order to get over these limitations [14]. Owing to the fact that transethosomes have a higher drug penetration across the skin into subcutaneous tissues and a higher entrapment efficiency than other vesicular carriers such as niosomes and liposomes, their usage in topical systems for drug delivery is growing [15]. Due to the transethosomes’ small size and ability to distort the skin obstruction and subsequently pass through the skin layer’s confined intercellular space intact, they have a greater potential for drug administration. The high ethanol content permits the transethosomes to disrupt the lipid bilayer, which makes the skin more malleable and makes it easier for drugs to be absorbed [16]. In addition to transethosomes’ deformability, its high content of ethanol permits the lipid membrane to organize less densely than in ordinary vesicles without impacting the structures’ durability and elasticity [17]. Moreover, transethosomes components are non-invasive, safe, and now authorized for use in pharmaceuticals [18].

Transethosomes can entrap a variety of lipophilic, lipophobic, and amphiphilic medications, peptides, proteins, and other active medicinal components [19]. It is possible to administer these transethosomes that are drug-entrapped in semisolid dosage forms like gel and cream. In cases of fungal infections, topical therapies are suggested as opposed to systemic ones. Transethosomes have been discovered to offer a potential advantage for the topical and transdermal delivery of antifungal medications for localized fungal infections of the skin. Drugs can be given directly to the area of localized infections using topical preparations in a controlled drug delivery profile. The drug can avoid the hepatic first-pass metabolism since it is not absorbed into the systemic bloodstream from the gut. Patient compliance may additionally be enhanced by providing a less complicated drug distribution route [20].

Based on our previous study, MCNR-loaded transethosomes (MCNR-TEs) were prepared and physicochemically characterized [21]. MNTG was safe and a non-irritant, and exhibited an increased in vitro antifungal activity against *Candida albicans*. Therefore, the aim of this study was to investigate the antifungal efficacy of MNTG against *Candida albicans* in an in vivo rat model. Histological examinations and the in vivo therapeutic effectiveness of the formulations were evaluated.

## 2. Results and Discussion

### 2.1. Patch Test (Skin Irritancy Test)

Pharmaceutical products are usually administered topically. A variety of skin reactions such as itchiness or allergic reactions might happen including redness, edema, leaking, scaling, and crusting. When developing a pharmaceutical product, it is important to ensure that all of its ingredients are safe to use on the skin [22]. One experimental in vivo method for determining a pharmaceutical product’s capacity to irritate the skin is the 24 h patch test [23]. It has also been shown that a patch test works better to anticipate skin sensitization reactions than a visual estimation of the inflammatory response [24]. The investigation on skin irritation was executed on healthy albino rats.

By performing skin irritation tests on healthy Wistar albino rats (females), the potential for skin irritation of MNG, MNTG, and the marketed cream (Daktarin^®^ cream 2%) was identified. The skin irritancy potential was evaluated by observing the progression of edema and erythema on rats’ skin. The primary cutaneous irritancy index score for MNG, MNTG, and the marketed cream (Daktarin^®^ cream 2%) was analyzed as shown in Table 1.

**Figure 1:**
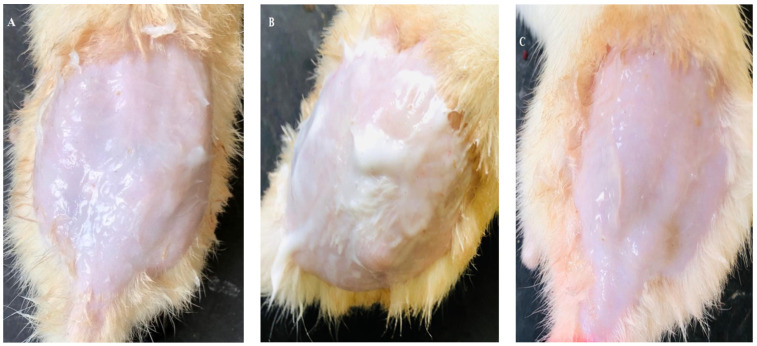
Rats’ skin after application of MNG (**A**), marketed cream (**B**), and MNTG (**C**).

According to skin irritation test, the transethosomal gel formulation was harmless to rat skin. On the rat’s skin, erythema and edema started to appear. The rats in group V (treated with MNTG) did not exhibit any serious erythema or edema development symptoms over the entire 48 h period. The rats in group III (treated with MNG) displayed severe edema and moderate erythema signs after 48 h. However, rats in group IV (treated with the marketed cream) showed minor edema. The primary cutaneous irritancy index scores for MNG, the marketed cream (Daktarin^®^ cream 2%), and MNTG were determined to be 2.9, 0.9, and 0.6 respectively, as demonstrated in Table 1. The rat skin after the application of MNTG, MNG, and the marketed cream is shown in Figure 1.

The data were subjected to a paired sample *t*-test and a two-way ANOVA. A significant difference between MNTG, MNG, and the marketed cream was observed. Compared to a usual irritant, MNTG’s irritancy score was significantly reduced (*p* ≤ 0.05).

This may be due to intact vesicles penetrating deeply and reducing miconazole nitrate molecules’ direct contact with skin. In other words, the nanocarriers shielded the skin from miconazole nitrate’s direct cutaneous contact. It may be concluded that the formulated MNTG is less of an irritant, well-tolerated, and secure for skin application.

### 2.2. Treatment Effect of the Gels

**Figure 2:**
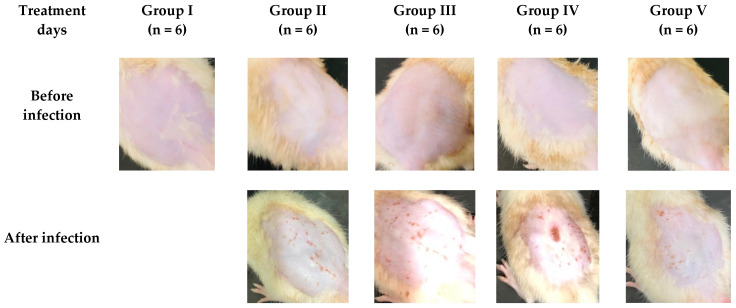

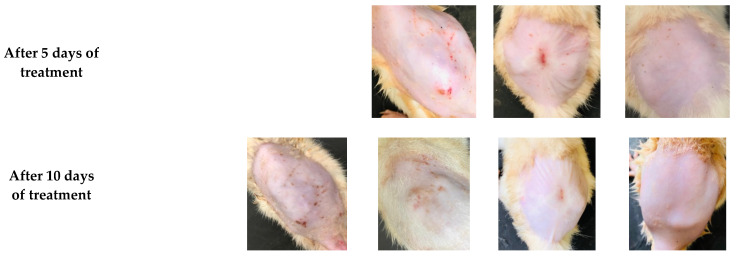
Rats’ skin before and after induction of fungal infection and after treatment with MNG gel (**Group III**)**,** marketed cream (Daktarin^®^ cream 2%) (**Group IV**), and MNTG (transethosomal gel) (**Group V**) for 10 days. **Group I** showing the rats’ skin of normal animals without any infection. **Group II** showing the rats’ skin after induction of infection without any treatment.

The assessment of an antifungal’s efficacy is frequently carried out using *Candida albicans* [25]. Figure 2 demonstrates the difference in animal skin before and after inducing a fungal infection, as well as a comparison of the skin following the treatment with MNG gel, the marketed cream (Daktarin^®^ cream 2%), and MNTG (transethosomal gel) for 10 days.

All animals displayed a normal skin structure prior to the induction of a cutaneous fungal infection, devoid of any clinical signs of fungal infection, such as swelling, edema, color changes, or cracking, as illustrated in Figure 2. The animals displayed red patchy areas, inflammation, scaling, edema, and skin cracking after the induction of the fungus. The infected rats were examined daily for indications of infection. The day after the subsequent instillation of the *Candida albicans* suspension in all of the animals, the first symptoms of infection in the form of skin redness were noticed. On the third day, it was more pronounced as scaling and obviously distinct skin redness.

After five days of treatment, the infection site showed signs of shedding of the infectious scales, revealing skin that was light pink in color. There were no significant changes in the structure of the skin after MNG gel treatment. The edema and inflammation accompanying the irritation decreased after 10 days of treatment with the marketed cream (Daktarin^®^ cream 2%), but the scars still remained. The MNTG, on the other hand, demonstrated normal skin after 10 days of treatment, as shown in Figure 2.

When compared to other formulations, the treatment for a fungal infection using the MNTG demonstrated a significant reduction in fungal infection, signifying that it is a more effective remedy. The outcomes of an in vivo antifungal trial revealed that the MNTG was more effective in eradicating *Candida albicans*-induced infections and minimizing its symptoms. This might be due to the drug’s increased ability to pierce deeper layers of skin, increasing its capability to remain in the skin. Drug retention in the skin is also influenced synergistically by the presence of the edge activator (oleic acid). The combined effects of the edge activator and ethanol, which function as permeation enhancers, increased the effectiveness of permeation even more. In contrast, due to the non-flexibility of the gel formulation, it is restricted to the *stratum corneum* surface layers and does not penetrate deeper areas of skin [20].

According to these findings, the antifungal activity of the formulations studied above is in the following order: Transethosomal gel (MNTG) > Marketed cream (Daktarin^®^ cream 2%) > MNG gel.

### 2.3. Histological Examination

The histopathological analysis is another aspect that provides proof for the test of antifungal activity. Similar to this research study, Zhang et al. employed the H&E approach to evaluate the effectiveness of the treatment they used in their investigation of skin wounds [26]. In this investigation, like in Wahedi et al. [27], the histological aspects were evaluated using H&E for the improved condition of skin tissue following a cure for a fungal infection. All five groups’ slides were examined using a compound microscope.

**Figure 3:**
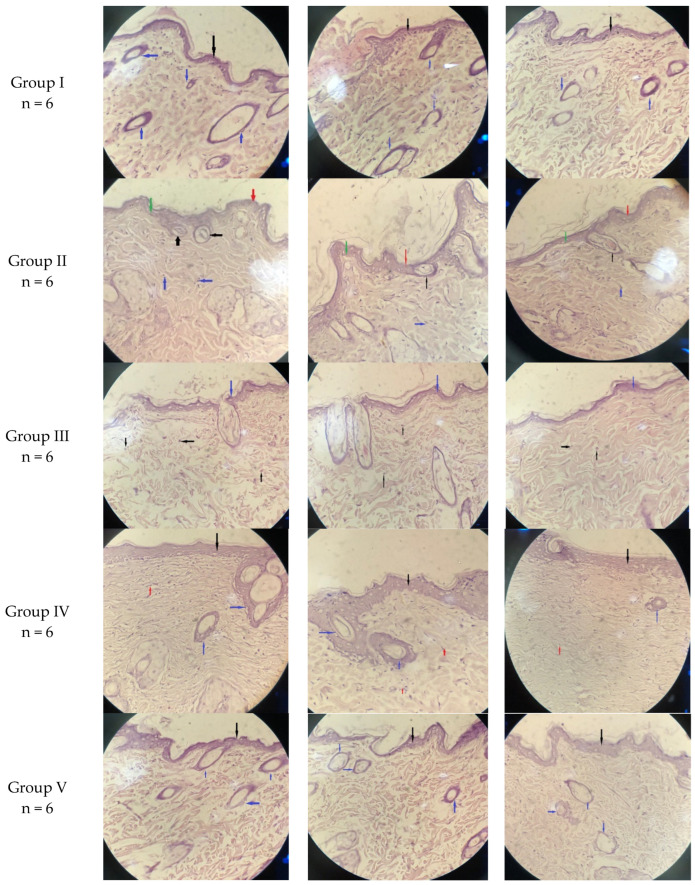
Histopathological images of rats’ skin of **Group I** (non-infected + normal), **Group II** (infected + untreated), **Group III** (infected + treated with MNG), **Group IV** (infected + treated with marketed cream), and **Group V** (infected + treated with MNTG) at magnification 40×.

A histopathological examination was carried out on healthy albino rats’ skin (negative control) and infected rats’ skin. The histological images of group I (negative control, non-infected), group II (positive control, infected, without receiving treatment), group III (infected and treated with MNG), group IV (infected and treated with the marketed cream), and group V (infected and treated with MNTG) are shown in Figure 3.

The negative control group (group I), as anticipated, presented a normal histological texture of the skin in the stained areas in 10 days of investigation. The dermis and epidermis layers of the skin of normal animals (Group I) were even, with no changes to the skin’s natural structure (marked with blue and black arrow). The blood vessels, collagen bundles, hair follicles, and sebaceous units were all visible in their usual sizes and shapes on the stained sections of a normal rat. Second, the infection-induced group (group II) experienced various pathological incidents, such as focal acanthosis (green arrow) with a trivial compact hyperkeratosis layer (red arrow), during the course of the 10 days but showed no recovery. The dermis layer had extensive, chronic inflammation, and the infiltration of inflammatory cells and the necrotic tissue region at the same moment as fungal hyphae appeared in the overlying epidermal layer (black arrow); and focal interface dermatitis (marked by blue arrow) also became visible. Third, after 10 days of treatment, the MNG-treated group (group III) exhibited only a very minor improvement. They demonstrated a slow recovery to normal skin morphology but did not completely eliminate inflammatory cells and recover the normal skin structure, as illustrated in Figure 3. The dermis and epidermal layer improved over the course of 10 days in the fourth group (group IV), which received treatment with the marketed cream (Daktarin^®^ cream 2%), with the exception of the skin appendages and focal acanthosis in H&E-stained segments.

Fifth, in the MNTG-treated group (group V), the skin tissues were fully recovered and showed considerable improvements in terms of rehabilitation from fungal infection. Group V demonstrated full restoration, a nearly normal tissue appearance, and a uniform morphology of skin in the dermis and epidermis layers, which may be associated with the transethosomes’s ability to penetrate multiple skin layers due to their nano-size to speed up the treatment of a fungus infection [28,29]. Moreover, edge activators’ inclusion in the chemical makeup of transethosomes improves skin penetration by making the transethosomal lipid bilayer more fluid and, thereby, making it easier for them to squeak into the skin pores [30,31]. Additionally, the high ethanol content increases skin permeability through two different processes: first, it intermingles with *stratum corneum* lipid molecules, altering their packaging of skin lipids and resulting in an upsurge in their permeability and fluidity; second, it makes transethosomal lipid bilayers more flexible and enhances their fluidity, which improves their ability to penetrate skin [32]. This enables a great permeability of MCNR in the case of MNTG, in contrast to the marketed cream (Daktarin^®^ cream 2%) and MNG, which contain free drug and have a low ability to penetrate the skin [33].

### 2.4. Hematological Analysis of Total and Differential WBC Count

To ascertain the test animals’ immunological condition, a hematology investigation was performed. In normal, infected, and treated groups of rats, the total and differential counts of WBC were assessed.

In the current study, the treated group’s mean WBC count was significantly higher than that of the untreated group, as demonstrated in Table 2. It is tempting to speculate that the MNTG protects rats from *Candida albicans* infection since the elevated WBC count suggests a normal state and indicates the absence of infection. In the differential count, lymphocytes were higher in number in the treated group than in the untreated group. When compared to the untreated and infected groups of rats, the lymphocytes were more abundant in the treated and normal groups of rats. After immunosuppression with prednisolone (5 mg/kg body weight), the lymphocytes in the untreated and infected groups of rats drastically decreased. The eosinophil count, however, displayed a contrary trend, as represented in Table 3.

The average gap between cells in the skin, according to Kaur et al., 2017b [34] and Kaur et al., 2017 [35] is 70 nm. Typical semisolid therapies, such as creams and lotions, permeate the skin more slowly. Transethosomal gel instantly penetrates the skin, delivering the active ingredients deeper and quicker, treating the infection, and normalizing the skin condition [34,35].

## 3. Materials and Methods

### 3.1. Materials

MCNR was provided by ATCO Laboratories Limited, Karachi, Pakistan. Daktarin^®^ cream was bought from Fazal Din Pharmacy (FDP), Bahawalpur, Pakistan. Oleic acid, triethanolamine, methanol, chloroform, and ethanol were purchased from Sigma Aldrich (St. Louis, MO, USA). Lecithin was a gracious present from Lipoid GmbH (Ludwigshafen, Germany). Sabouraud dextrose agar was obtained from Thermo Scientific TM (Waltham, MA, USA). Carbopol-934 was acquired from Merck KGaA (Darmstadt, Germany). Formaldehyde was provided by DAEJUNG, Ltd. (Siheung, Republic of Korea). Double-distilled water was manufactured using a Milli-Q Gradient A10 System (Millipore, Billerica, MA, USA) in the Pharmaceutics Lab of Islamia University of Bahawalpur, Pakistan. *Candida albicans* with *ATCC 10231* was procured from LABNOSTIX LTD (Warrington, UK).

### 3.2. Preparation of Miconazole Nitrate (MCNR) Transethosomal Formulation

Miconazole-nitrate-loaded transethosomes (MCNR-TEs) were prepared using the conventional thin-film hydration method with the same conditions employed in our previous study [21]. Briefly, in a dry round bottom flask, MCNR (100 mg) was added together with 85 mg of lecithin and 15 mg of oleic acid in a 3:1 mixture of chloroform and methanol. The organic solvents were vaporized through a rotary evaporator (Heidolph, Kelheim, Germany) set to 60 rpm, under low pressure, and, at 55 °C, a thin lipid film was created on the wall of the round-bottom flask. The film was then hydrated using a phosphate buffer solution (pH 5.5) and ethanol for 1 h at 100 revolutions per minute at room temperature. The preparation was then sonicated for 15–20 min to reduce the size of the particles before being stored at 4 °C for further research [25].

### 3.3. Preparation of Transethosomal Gel and MCNR Gel

The transethosomal gel containing miconazole nitrate (MNTG) was formed using 1.5% *w*/*w* carbopol-934 as a gelling agent [36]. Triethanolamine (0.05% *w*/*w*) was added dropwise with continuous homogenization until a transparent gel with a pH range of 5.5 to 6.5 was produced. The MCNR-TEs was smoothly added to the gel while being uninterruptedly mixed with a homogenizer to produce a homogenous gel formulation including transethosomal vesicles [21].

The identical procedure as described above was used to fabricate miconazole-nitrate-loaded gel (MNG), with the exception of transethosomal formulation being replaced with a simple solution of miconazole nitrate. MCNR was first dissolved in a small amount of methanol to produce the miconazole nitrate solution, and then a homogenizer was used to slowly add the methanolic solution to the gel [37].

### 3.4. In Vivo Evaluation of MCNR Transethosomal Gel

#### 3.4.1. Animals

The in vivo study was conducted using female Wistar albino rats weighing (110–130 g). Animals were purchased from a local market in Bahawalpur, Pakistan. They were kept in the Experimental Zone 2 Animal House in the Pharmacology and Physiology Research Laboratory at the Islamia University of Bahawalpur, Pakistan. The procedures used comply with the ethics and rules of the UK Animals (Scientific Procedures) Act of 1986. The Pharmacy Animal Ethics Committee at the Faculty of Pharmacy, The Islamia University of Bahawalpur in Pakistan gave its approval to all methods. Animals were appropriately housed in polycarbonate cages with open access to water and a standard diet comprising all necessary nutrients in conventional laboratory settings. The animals were kept in typical laboratory environments, which included a temperature of 25 ± 2 °C. The cycle of twelve hours of light and twelve hours of darkness was well kept-up, and artificial light was given [38].

#### 3.4.2. Experimental Design

The Pharmacy Animal Ethics Committee of the Islamia University of Bahawalpur in Pakistan approved the current study. The issued registration number was PAEC/23/94. Five groups were used to conduct this study. Each group contained six (n) rats and were randomly divided as follows:

Group I served as the negative control group that was fungus-free (without fungal infection);

Group II served as the positive control (fungal infected), without receiving any treatment (untreated);

Group III included infected animals that underwent 10 days of topical treatment with miconazole-nitrate-loaded gel (MNG);

Group IV included infected animals that underwent 10 days of topical treatment with marketed cream (Daktarin^®^ cream 2%);

Group V included infected animals that underwent 10 days of topical treatment with miconazole-nitrate-loaded transethosomal gel (MNTG).

#### 3.4.3. Patch Test (Skin Irritancy Test)

Before beginning the study, a 48 h irritation test was conducted on the rats’ skin to test them for any sensitivity. This study was carried out for acute cutaneous irritation and corrosion in accordance with the Organization for Economic Co-operation and Development (OECD) test guidelines 404. Rat hairs (4 cm^2^) were removed 24 h before the experiment from the experimental site using a hair removal cream. In this test, the rats from groups III, IV, and V received applications of MNG, marketed cream (Daktarin^®^ cream 2%), and MNTG, respectively. Approximately 0.5 g of each test product was topically administered to the skin’s surface for each group of rats. After 24 h, the test samples were removed, and distilled water was used to clean the skin’s surface. The following day, at 0 h, 24 h, and 48 h, the experimental sites were examined for skin irritation [39,40]. The primary cutaneous irritancy index was calculated by combining the erythema and edema scores for each group. Scores for the erythema ranged from 0 to 4, with 0 denoting no erythema or edema and 1, 2, 3, and 4 denoting very slight, slight, moderate, and severe erythema/edema, respectively. The observations were recorded as numerical scores for each animal [41]. The mean scores for erythema and edema, noted to be relying on the degree of erythema/edema, were as follows: no erythema or edema = 0, very slight erythema or edema = 1, slight erythema or edema = 2, moderate erythema or edema = 3, and severe erythema or edema = 4.

The MNTG scores were then compared to MNG and marketed cream scores.

#### 3.4.4. Immunosuppressed Animal Preparation

Prior to being assigned to the experimental regimen, all animals were given a week to acclimate under conventional animal house conditions. To test the effectiveness of the MNG, MNTG, and marketed cream (Daktarin^®^ cream 2%) in curing deeper fungal infections, immunosuppression was used to create an intense skin infection. Prednisolone (5 mg/kg) given intravenously to rats for three days resulted in an immunosuppressive state before a fungus infection was introduced [42].

#### 3.4.5. Fungal Strain Preparation

*Candida albicans* was grown in sabouraud dextrose agar for 48 h at 35 °C in order to cause cutaneous candidiasis in Wistar albino rats. Following the collection of yeast colonies and their suspension in sterile saline, the final concentration of *Candida albicans* was adjusted to give 10^6^ CFU/mL [33]. According to the Clinical Microbiology manual, the concentration of the cell suspensions was evaluated using a spectrophotometric approach by detecting their turbidity at 530 nm [43].

#### 3.4.6. Development of Fungal Infection

Each rat received an intradermal injection of 0.3 mL of a *Candida albicans* suspension containing 10^6^ CFU/mL in the middle of a shaved area on its exposed skin. The small amount of edema on the injection site was removed by vigorously rubbing it. After 72 h, the fungal infection was discovered in the suffering area [1,33]. To avoid skin-licking, the animals were kept separately in their own cages.

#### 3.4.7. Clinical Examinations

In order to spot any clinical signs of a fungal infection, all rats were monitored both before and during the performance of tests. Rashes, red patches, white particles, scaling, maceration, erythema, cracking, and pus-filled pimples were among the symptoms that were seen and noted [1].

#### 3.4.8. Histopathological Analysis

Rats were sedated before being sacrificed at the end of the studies. Ketamine was utilized as an anesthetic. A 1:10 mixture of ketamine 0.2 mL/100 mg and xylazine 50 mg/kg is used. Skin from the affected area was taken out, and secured with 10% formalin, and then clogged with paraffin. Slides made from paraffin blocks with the use of a rotary microtome (Bright Instruments 5040 Microtome, Hntingdon, Cambridgeshire, UK), were divided into 5 µm-thick cuts [44] and stained with hematoxylin–eosin dyes [45,46]. The assessment of the various animal groups was carried out, and the outcomes were compared to control groups. Using a compound microscope (Leica Microsystems, Wetzlar, Germany), the skin samples were examined to identify the epidermal and dermal modifications as well as the signs of inflammation [47].

#### 3.4.9. Assessment of Blood Parameters

Blood was obtained in a glass vial containing anticoagulant (EDTA) on the tenth day following the animals’ sacrifice in order to analyze the hematological parameters. A standard technique was used to determine the total and differential counts of white blood cells (WBC) in normal, infected, and treated groups of rats with Countess 3 Automated Cell Counter (Thermo Fisher Scientific, Waltham, MA, USA) [48].

### 3.5. Statistical Analysis

The study’s findings were assessed statistically and scientifically using IBM SPSS version 23. A two-way analysis of variance was conducted to determine variations in the findings. The *p*-values necessitate being less than 5% (*p* ≤ 0.05) for the results to be deemed statistically significant [49,50]. To analyze the total WBC count, (mean *±* SD) were used. Student *t*-tests were used to analyze the total WBC count (mean ± SD), and an ANOVA one-way test was employed to analyze the differential count (at 5% significant level).

## 4. Conclusions

Miconazole nitrate (MCNR) transethosomes were successfully produced via the thin-film hydration approach and incorporated into carbopol-934 base gel to form transethosomal gel. The MNTG demonstrated notable therapeutic success in the treatment of *Candida albicans*-induced cutaneous candidiasis. Compared to the marketed cream (Daktarin^®^ cream 2%) and MNG, the MNTG demonstrated enhanced antifungal efficacy against *Candida albicans*, with no signs of irritation, which confirmed the safety of MNTG for skin application. The study’s findings indicate that the MNTG can be used as a topical drug delivery system with improved antifungal efficacy. This suggests a way to get around the higher doses of MCNR needed for distinctive topical administration, systemic side effects, and frequently received applications. Consequently, MNTG has the potential to be a carrier for the topical treatment of fungal infections.

## Figures and Tables

**Table 1:** Comparison of primary cutaneous irritancy index after application of MNG, marketed cream, and MNTG.

Time (h)	MNG		Marketed Cream		MNTG	
	Erythema	Edema	Erythema	Edema	Erythema	Edema
0 h	0	0	0	0	0	0
24 h	1	1	1	0	1	1
48 h	3	4	0	2	0	0
Mean ± SD	1.3 ± 1.57	1.6 ± 1.20	0.3 ± 0.21	0.6 ± 0.18	0.3 ± 0.04	0.3 ± 0.05
Primary cutaneous irritation index	2.9		0.9		0.6	

Abbreviations: MNG, miconazole nitrate gel; MNTG gel, miconazole nitrate transethosomal gel.

**Table 2:** White blood cell (WBC) counts in normal, infected, and treated groups (mean ± standard deviation, n = 6).

Subject	White Blood Cells/mcL
Group I (normal)	9800 ± 145
Group II (infected + untreated)	4300 ± 265
Group III (infected + treated with MNG)	6700 ± 318
Group IV (infected + treated with marketed cream)	8800 ± 256
Group V (infected + treated with MNTG)	9500 ± 240

Note: The values of six animals in each group are expressed as the mean ± standard deviation (significant at level of 5%).

**Table 3:** Differential white blood cell (WBC) counts in normal, infected, and treated groups (mean ± standard deviation, n = 6).

Subject	Lymphocytes (%)	Eosinophils (%)
Group I (normal)	43	1
Group II (infected + untreated)	15	6
Group III (infected + treated with MNG)	30	3
Group IV (infected + treated with marketed cream)	37	2
Group V (infected + treated with MNTG)	41	2

Note: The values of six animals in each group are expressed as the mean ± standard deviation (significant at level of 5%).

## Data Availability

The corresponding author can provide the data described in this study upon request.
